# Portuguese translation, cultural adaptation, and validation of the Person-Centered Practice Inventory – Care

**DOI:** 10.1371/journal.pone.0324286

**Published:** 2025-05-28

**Authors:** Diana Vareta, Célia Oliveira, Brendan McCormack, Paul Slater, Vaibhav Tyagi, Filipa Ventura

**Affiliations:** 1 PhD Program, University of Lisbon (UL) and Nursing School of Lisbon (ESEL), Lisbon, Portugal; 2 Egas Moniz Interdisciplinary Research Centre (CiiEM), Egas Moniz Universitary Institute, Monte de Caparica, Portugal; 3 Nursing School of Lisbon (ESEL), Lisbon, Portugal; 4 The Susan Wakil School of Nursing and Midwifery, The University of Sydney, Sydney, Australia; 5 School of Nursing, Ulster University, Jordanstown, UK; 6 The Health Sciences Research Unit: Nursing (UICISA:E), Nursing School of Coimbra (ESEnfC), Coimbra, Portugal; School of Nursing Sao Joao de Deus, Evora University, PORTUGAL

## Abstract

**Background:**

In person-centered practice implementation and development, it is essential to incorporate standardized measurements that consider the perspectives of those involved in the therapeutic relationship. This work aims to translate, culturally adapt, and validate the Person-Centered Practice Inventory – Care (PCPI-C) for the Portuguese healthcare context. The PCPI-C is derived from the middle-range theory of the Person-Centered Practice Framework and is an 18-item self-reported inventory.

**Methods:**

This methodological study followed a two-stage research design entailing the translation and cultural adaptation of the PCPI-C from English to European Portuguese and the Portuguese healthcare context in phase I, followed by a psychometric evaluation (N = 312) conducted using principal component and confirmatory factor analysis in SPSS version 27.0 and SPSS AMOS version 21.0 in phase II. The model was continuously and iteratively refined until it was considered acceptable per gold standard estimators.

**Results:**

In phase I, the results revealed linguistic and contextual cultural differences compared to the original version. The cognitive debriefing showed that the respondents considered the items understandable and adequate for the purpose. In phase II, among the two adjusted PCPI-C models fit, i.e., first-order, and single-factor, the best fit to the empirical data was revealed by the single-factor structure, reflecting a good fit (x2/df = 2.408, CFI = .93, RMSEA = .07, SRMR = .05).

**Conclusions:**

The PCPI-C is a valid and reliable instrument for assessing the perceptions of Portuguese service users regarding person-centered practice. It is necessary to consider the purpose for which the instrument is used to select the most appropriate measurement model, i.e., process evaluation as an outcome or as an inventory measure for continuous improvement.

## 1 Introduction

Person-centered care is recognized as a fundamental approach to the quality, safety, and sustainability of healthcare services in the face of an aging trend, the increasing prevalence of chronic diseases, and the growing complexity of health needs [1,2]. Person-centered care has been underpinning the development of new health policies and strategies at the international level [1–3]. This approach to care values the person’s participation in the healthcare relationship, supports shared decision-making and mutual understanding, respects a person’s values, preferences, and beliefs, meeting the health and social needs of the population throughout the life course [1].

The Person-centered Practice Framework (PCPF) developed by McCormack and McCance [4] is an internationally recognized theoretical framework that assists multidisciplinary teams in gaining a better understanding of the domains of person-centeredness and provides guidance to operationalize it in practice. This framework was grounded in a middle-range theory that integrates the fundamental domains of person-centered care and covers all levels of care provision (i.e., individual, organizational, and structural levels), constituting a reference for its implementation and development [4,5]. The domains include ‘prerequisites’, ‘the care environment’, ‘person-centered care processes’, and ‘the macro context’ of the healthcare system. Along with the development of the PCPF, the foundations and definitions of person-centered practice have become increasingly consistent and consensual, overcoming some implementation barriers identified in the literature [5–7]. The challenge, however, continues to be the way through which these principles are translated into everyday practice to enable multidisciplinary teams to consistently deliver this standard of care over time [8–10]. There is a recognized need to produce evidence based on the effectiveness of person-centered care through measurement instruments that have a sound underpinning on a theoretical framework for this purpose [11–13]. The evaluation should further consider diverse perspectives (i.e., service users, staff) and approaches (e.g., questionnaires, observations) to ensure a comprehensive assessment of the person-centered practice [3,8,14]. To validate the implementation and monitor the development in practice, authors have derived inventories from the PCPF that examine the way through which the person-centered practice is perceived.

Since the first version of the PCPF in 2006 [15], the framework has evolved in line with emerging research and evidence, particularly regarding multidisciplinary team perceptions since the release of the Person-Centered Practice Inventory - Staff (PCPI-S) [8,16,17]. Aware of the need to consider the perspectives of those involved in the therapeutic relationship, the authors of the PCPF further develop an inventory to gather the perspectives of the service users on the person-centered practice, Person-Centered Practice Inventory – Care (PCPI-C) [18].

In Portugal, research on person-centered practice in the daily care practice of healthcare departments started with the application of the PCPI-S [19,20]. However, the perception of the person being cared for about the person-centered practice in Portugal has yet to be uncovered, and an adequate measurement instrument that is underpinned on a person-centered theoretical basis is not available. Therefore, this study aims to translate and culturally adapt the PCPI-C to the Portuguese healthcare context and evaluate its psychometric properties.

## 2 Materials and methods

### 2.1 Description of the PCPI-C

The original psychometric properties of PCPI-C were established in a multinational study involving participants from the UK, Portugal, Poland, Malta, and Austria (N = 452) [18]. The inventory demonstrated sound factorial validity, supporting a five-factor model of person-centred practice. The adjusted model showed improved fit (RMSEA = .053, CFI = .93, SRMR = .048), indicating an acceptable level of model adequacy. The correlation matrix showed item correlations ranging from .144 to .740, suggesting breadth across care domains and no evidence of collinearity. Internal consistency was supported by satisfactory Cronbach’s alpha coefficients, with all factor loadings statistically significant and above the acceptable criterion of .3 for the sample size. These findings confirm that the PCPI-C is a reliable and valid instrument for assessing service users’ perspectives on person-centred care. The PCPI-C is a 5-point Likert scale composed of 18 items that assess the patient’s level of agreement with statements about the ‘person-centered processes’ domain described in the PCPF, with higher values indicating greater agreement. Participants are asked to evaluate statements ranging from ‘strongly disagree’ (1) to ‘strongly agree’ (5). The domain is composed of five constructs, specifically, i) ‘working with patients’ beliefs and values’, ii) ‘sharing decision-making’, iii) ‘engaging authentically’, iv) ‘being sympathetically present’, and v) ‘working holistically’ [18].

The inventory enables healthcare services to explore service users’ experiences with the person-centered practice by inquiring about the process through which they were cared for across several healthcare settings [18].

### 2.2. Study design

This methodological study followed a two-stage research design entailing the translation and cultural adaptation of the PCPI-C from English to European Portuguese and the Portuguese healthcare context (i.e., phase I), followed by a psychometric evaluation with a cross-sectional quantitative approach (i.e., phase II).

The study was conducted following the Declaration of Helsinki and was approved by the Hospital Ethics Committee (ref. nr. 36/2021; date of approval: September 2021). Authorization for carrying out the translation and cultural adaptation was obtained from the original authors, with whom contact was kept throughout the study to enhance the methodological rigor and translatability between contexts.

#### 2.2.1 Phase I: Translation and cultural adaptation.

The translation and cultural adaptation of the original version of the PCPI-C into European Portuguese followed the principles of good practice in translation and cultural adaptation approved by the International Society for Pharmacoeconomics and Outcomes Research (ISPOR) [21] in a 10-step methodological procedure, with the last step being the elaboration of the phase I report.

The cognitive debriefing step aimed to check the comprehensibility, interpretability, and cultural relevance of the PCPI-C translation and was conducted according to Willis [22]. This step entailed the identification and recruitment of adults (i.e., age over 18 years) who wanted to participate on a voluntary basis in the process of translation of the inventory. The inclusion criteria were the ability to read and write fluently in Portuguese, having experienced with health care in hospital settings, and having different characteristics of interest for the research, namely, gender, age, and years of education. The recruitment for the cognitive debriefing was guided by the main principle of covering the spectrum of the characteristics under analysis rather than achieving statistical representation [23]. Potential participants received written information about the study in person and were invited to participate in an individual interview specifically developed for this study (S1 AppendixF). A face-to-face interview was scheduled with those who agreed to participate. Before starting, participants were given verbal information about the study that explained the nature of their participation. They were further informed that they could leave at any time without justification and were granted confidentiality and anonymity, as no personal identification was collected. During the individual cognitive interview, a form containing the informed consent, sociodemographic variables, and the harmonized version of the Portuguese PCPI-C was delivered. After providing written informed consent, the participants were instructed to complete the inventory independently, and subsequently, guidance on the reflective activity was provided. The participant was asked to comment on any aspect that he/she considered to have been omitted or that had raised doubts. The Think-Aloud method was used [22]. After that, specific questions concerning the inventory were asked through the verbal probing technique, which was applied retrospectively. In so doing, the participants expressed their understanding of each statement clearly and thoroughly. When the participant considered the item problematic (e.g., difficult to read, inadequate wording), the moderator asked for alternative formulations. All interviews were audio-recorded, transcribed verbatim, and analyzed for discrepancies and similarities in interpreting concepts across participants and cultural inadequacies [22].

#### 2.2.2 Phase II: Psychometric evaluation.

Quantitative cross-sectional research was conducted to generate a dataset for the psychometric validation of the Portuguese version of the PCPI-C. The inventory was tested in a convenient sample with the following inclusion criteria: aged 18 years or older, able to read and write in Portuguese, being hospitalized at the Internal Medicine Department for more than 48 hours or having received health care for more than 48 hours at the internal medicine department in the last six months, to reduce memory bias in response to self-reported instruments [24]. The recruitment period occurred between 03/03/2022 to 10/07/2023. In the internal medicine department, prospective participants were first given oral and written information about the study, including its purpose, relevance, data collection methods, expected participation, and disclosure of the information collected. Between the presentation and validation of understanding of the information provided and the start of data collection, a reflection period of at least 24 hours was ensured so that participants could consider their decision and complete the informed consent form. The inventory was made available to the participants in paper format for self-reporting. In case the participants had difficulties in self-completion, assistance was provided. In these cases, the principal investigator conducted the interview strictly following the PCPI-C questions, ensuring the confidentiality of the information collected and without adding any elements that would allow the participants to be identified. The data were collected as close to discharge from the department as possible to ensure sufficient knowledge of the subject under study and to restore the participant’s well-being.

Participants with a history of hospitalization in the last six months were recruited from the Internal Medicine Day Hospital. This unit follows patients after an episode of hospitalization in the Internal Medicine Department. Accordingly, the probability of finding people meeting the inclusion criteria would be higher. The nurses working at the Internal Medicine Day Hospital identified the eligible participants according to the inclusion and exclusion criteria. After that, the participants were contacted by phone, informed about the scope of the study, and invited to participate. Those who agreed were asked for oral consent to share their names and to schedule an interview with the principal investigator. On the day of the interview, oral and written information about the study was provided by the principal investigator, namely its purpose, relevance, data collection methods, expected participation, dissemination of the information collected, right to withdraw from the study at any time without any repercussions, and right to access the study findings by contacting the principal investigator. Anonymity and confidentiality were assured at this stage since no personal elements that could act as identifiers were collected from respondents. Subsequently, informed consent was obtained, and the participants were given a form that included the translated and culturally adapted PCPI-C and sociodemographic variables, namely gender, age, and length of stay. The estimated time required to complete the inventory is approximately 10 minutes.

### 2.3 Data analysis

The statistical package for social sciences software SPSS statistics version 27.0 and SPSS AMOS version 21.0 were used to conduct the data analysis (IBM SPSS Statistics^®^ for Windows, IBM Corp. Released 2020, Armonk, NY, USA). Descriptive statistics of the respondents’ characteristics and item statistics were generated. Missing data analysis was performed. Floor and ceiling effects were examined, with values greater than ± 2 indicating the presence of skewness/kurtosis [25]. Kolmogorov‒Smirnov and Shapiro‒Wilk tests of normality were used to determine the distribution of the data.

Reliability analysis included internal consistency, using Cronbach’s alpha for the whole domain of ‘person-centered processes’ and individual composing constructs. Values between .70 and .79 were considered evidence of acceptable internal reliability, values between .80 and .89 indicated good internal reliability, and values of.90 or higher indicated excellent internal reliability [26].

Construct and structural validity were determined by item-construct and item-total correlations, principal component analysis (PCA) and confirmatory factor analysis (CFA). Correlations were judged as inadequate if < .20, sufficient if ranging from .20 –.34, good if ranging from .35 –.49 and excellent if ≥ .50 [26].

The Kaiser-Meyer-Olkin (KMO) measure of sample adequacy of .907 (≥ .6) and a significant Bartlett’s test of sphericity (✗^2^ (153) = 2482,11, *p* < .001), indicated adequacy for the data reduction procedures. PCA with orthogonal varimax rotation was used for extraction. The number of factors was identified by the Kaiser criterion of a eigenvalue ≥ 1 and a scree plot [25]. CFA was conducted for the PCPI-C corresponding to the PCPF ‘person-centered processes’ domain. The maximum likelihood estimation method recommended for categorical, nonnormally distributed data was used [27]. The outliers were determined through the Mahalanobis distance for each data case. The items’ factor loadings were examined, and the threshold for acceptability was defined based on the sample size achieved [28]. Correlated items with a modification index (MI) greater than 11 were reviewed to potential modification [28]. Modifications to the original structure of each domain were defined in congruence with the criteria used in previous validation studies of the PCPI-S in Norwegian [29], German [30] and Portuguese [19] in the following order: 1) correlated errors across items within the same factor, 2) correlated errors across items across factors, and 3) correlated factor loadings of items to factors. Modifications were only allowed if they were congruent with the theoretical framework. The instrument model was refined continuously and iteratively until it was considered to have acceptable fit. Following the recommendations from the literature, model fit was attained if at least one criterion from each component revealed acceptable fit [27,28]. In this study, the fitness indices used were the root mean square error of approximation (RMSEA; acceptable fit ≤ .06), root mean square residual (SRMR; acceptable fit < .08), Tucker-Lewis’s index (TLI; acceptable fit ≥ .9), comparative fit index (CFI; acceptable fit ≥ .9), chi-square (✗^2^)/degrees of freedom (df) (acceptable fit ≤ 5).

## 3 Results

### 3.1 Phase I: Translation and cultural adaptation

According to the ISPOR guidelines [21] the methodological procedure followed the steps described below.

1) *Preparation*: This step entailed the request for formal authorization from the PCPI-C authors, which was obtained in June 2021. The key concepts addressed in the inventory were identified and defined, and each item was linked to the corresponding construct of the PCPF [4].
2) *Forward translation*: Two independent translators were recruited, following the criteria of being bilingual native speakers of the target language and having experience with translating patient-reported outcomes measures in the healthcare domain for at least one of the translators.
3) *Reconciliation*: The Portuguese versions resulting from the direct translation were compared and reconciled into a single version after discussion with a third person of reference in person-centered care in Portugal. Identifying and defining the key concepts of the inventory and the reconciliation of the two forward-translated versions allowed the identification of linguistic differences related to the Portuguese culture and healthcare system. The following adjustments were made as a result of the reconciliation phase:

- Staff was translated as ‘health professionals’ in items 3, 4, 7, 11, 12, 13, 15, 16 and 17, maintaining the concept that refers to all professionals with legitimacy to provide health care [31]. As explained by Ventura et al. [19], “attaining the definition of the construct of ‘Appropriate skill mix’, the Portuguese healthcare system does not comprise staff working as non-registered nurses and the multidisciplinary designation of ‘staff’ was found to be ambiguous.”
- Caring was translated as ‘care’ in items 3, 4, 5, 7, 11, 12, 13, 15, 16 and 17 because it is the Portuguese wording most closely related to the concept described by the authors [4].
- In item 4, ‘home environment’ was translated to ‘home context’, as it refers not only to the practices and relationships among the cohabiting family members but also to the characteristics of the dwelling him/herself.
- In item 7, feedback was translated as ‘opinion’ because it is a more familiar term to the user. The alternative wordings of ‘evaluation’, ‘comment’, ‘point of view’, and ‘perspective’ were also considered. ‘Being cared for’ was translated as ‘care provided to me’ according to the need to adapt the verb form to the Portuguese language.
- In item 9, the expression ‘connect with me’ was harmonized to ‘establish a connection with me’ to ensure a discourse closer to the user.
- ‘Consensus’ was chosen as the translation of ‘common ground’ in item 11 because it is the term that most simply represents the concept of reaching a common agreement.
- In item 14, compassionately was harmonized with ‘compassion,’ as the term ‘compassionate’ is not used colloquially in the Portuguese language and ‘comprehensive’ departs from the original meaning.

4) *Back translation*: A new translation of the Portuguese version into the original language was performed by a native translator unfamiliar with the inventory’s content. The aim was to verify the quality of the translation and find conceptual equivalence in the version translated into the source language.
5) *Back translation review*: A comparative analysis was conducted to identify and manage discrepancies and ensure conceptual clarity between the original and the reconciled versions. The changes resulting from the comparative analysis between the back-translation and the original version came from a consistent approach to translation problems solved in the reconciliation phase.
6) *Harmonization*: This step was not carried out because there were no previous translated or published versions of the PCPI-C.
7) *Cognitive debriefing*: Interviews were conducted with a sample of seven people. The sample was mainly composed of women (71.4%). The participants’ ages ranged between 34 and 75 years, and their education levels ranged between 12 and 18 years. Each participant reviewed the 18 items of the inventory following the instrument structure, and the average length of the interviews was 36 minutes. The first round consisted of four interviews that focused on interpreting general concepts [32]. Overall, the cognitive debriefing revealed the interpretability and adequacy of the items. However, changes were made due to the ambiguity of interpretation among participants and the adaptation to colloquial discourse. Thus, in item 3, ‘decision making’ was replaced by ‘decisions,’ as the first term is more frequently used in professional settings; in item 7, ‘give my opinion’ was changed to ‘share my perception,’ as the term opinion was associated with a negative connotation by two of the interviewees and could be based on aspects other than the experience of care. In item 14, ‘compassion’ was associated with depreciative connotations by one of the participants. The concept was discussed with the PCPF authors, who refer to compassion as a strong feeling of sympathy and sadness for the suffering or bad luck of others and a wish to help them. Therefore, both ‘compassion’ and ‘empathy’ were tested in the next round. In the second round, with three interviews, adjustments were made to the flagged items, alternative spellings were tested, and orthographic, structural, and grammatical form issues were validated. Throughout the cognitive interview process, 15 of the 18 items remained stable from the first round, item 3 was linguistically adjusted to simplify the interpretation, and items 7 and 14 were culturally adapted while maintaining conceptual equivalence.
8) *Review of cognitive debriefing results*: With the changes resulting from the cognitive interviews, the version obtained was compared with the original version to check whether the interpretation of the items remained equivalent. As a result, the research team validated the conceptual and cultural equivalence of the final version.
9) *Proofreading*: review to correct typographic, grammatical, or other errors.

## 3.2 Phase II: Psychometric evaluation

A sample of 312 participants with recent experience as healthcare service users answered the Portuguese PCPI-C. The sample was comprised of 49.4% women and 50.6% men, with 80.8% of all participants being older than 65 years (M = 71.9; SD = 10.8; Min = 28; Max = 93). The length of stay ranged from 2 to 93 days (M = 8.13; SD = 8.71), with 51.3% of participants staying up to 6 days, 32.7% between 7 and 9 days, and only 16% more than 10 days (Table 1).

**Table 1:** Sample characteristics.

Variable	N	%
*Gender*		
Female	154	49,4
Male	158	50,6
*Age*		
25-35	2	.6
36-45	7	2.2
46-55	16	5.1
56-65	35	11.2
66-75	131	42
76-85	94	30.1
≥ 86	27	8.7
*Lenght of stay*		
2-3	30	9.6
4-6	130	41.7
7-9	102	32.7
≥ 10	50	16

Considering that the PCPI-C measures one domain, i.e., ‘person-centered processes’, a 5:1 ratio of factorial analysis was achieved [30,33,34].

Missing data analysis revealed no missing data for the 18 items. All items were scored positively, with the mean ranging from 3.26 (item 8) to 4.38 (item 5).

Skewness was not verified in the 18 items, and kurtosis was identified for items 1, 2, 5, 6, 7, 9, 12, 13, 15 and 16, indicating a leptokurtic distribution and the presence of ceiling effects (Table 2) [25]. However, these scores are lower than 7. No relationship was verified between kurtosis and the construct to which each item belonged. Normality of the sample distribution was not present (Shapiro-Wilk test p < .05).

**Table 2:** Mean scores, measures of distribution and factor loadings of each item of the PCPI-C (PT).

Construct	Mean	Sd	Skewness	Kurtosis	Cronbach alpha
Person-centered processes					.90
*Working with the Person’s Beliefs and Values*					.69
1. Healthcare professionals make an effort to understand what is important to me	4.34	.62	-1.35	6.27	
6. I feel free to say healthcare professionals what is important to me	4.25	.67	-1.19	3.46	
7. I feel capable to share my perceptions about my care experience with healthcare professionals	4.08	.69	-.95	2.76	
12. In caring for me, healthcare professionals use what they know about me as a person	3.98	.78	-1.51	3.76	
*Sharing Decision-making*					.57
3. Healthcare professionals involve me in decisions about my care	4.01	.76	-.98	1.56	
10. Healthcare professionals ask me if I have all the information I need	3.58	1.08	-.62	-.53	
15. Healthcare professionals help me to express my concerns about my treatment and care	4.10	.68	-.94	2.14	
18. Healthcare professionals help me to define realistic goals	3.92	.83	-.98	1.16	
*Engaging Authentically*					.62
9. Healthcare professionals establish a connection with me as a person	4.12	.67	-1.37	5.00	
11. When we disagree about my care, healthcare professionals try to reach a consensus	3.78	.72	-.23	-.09	
16. Healthcare professionals listen to me and hear what I have to say about care	4.17	.66	-1.15	3.68	
*Being Sympathetically Present*					.62
2. Healthcare professionals use my personal experiences to build a relationship with me	3.99	.75	-1.34	3.32	
5. Healthcare professionals give me their full attention when they are with me	4.38	.59	-.91	3.44	
14. Healthcare professionals respond with compassion when I am upset or unhappy	3.92	.80	-.74	.66	
*Working Holistically*					.71
4. Healthcare professionals take my home context into consideration in satisfying my care needs	3.75	.99	-.79	-.04	
8. Healthcare professionals ask me questions about my life	3.26	1.16	-.37	-1.10	
13. I feel that I’m cared for	4.36	.57	-.72	3.31	
17. Healthcare professionals understand my family circumstances when they take care of me	3.77	.92	-1.04	.80	

The Cronbach’s alpha score for the total inventory was excellent (α = .90). Furthermore, the constructs ‘working with the person’s beliefs and values’ (α = .69), ‘engaging authentically’ (α = .57), ‘being sympathetically present’ (α = .62), ‘sharing decision making’ (α = .74) and ‘working holistically’ (α = .71) revealed acceptable internal consistency.

The item-construct correlation was excellent, ranging from .58 to .81, and the item-total correlations varied between good for item 11 (.49) and excellent for all the other 17 items.

An examination of the correlation matrix showed scores ranging from a nonsignificant relationship of .14 to .64, confirming the absence of collinearity (S1 Table).

PCA with orthogonal varimax rotation revealed three components aligned with an eigenvalue ≥ 1. These factors accounted for 55.3% of the variance explained, which is considered adequate as they allow to capture a significant portion of the variability in the data (S2 Table).

In keeping faithful to the theoretical structure of the inventory, a CFA was conducted to test the predetermined model of five components, thereby validating the structure in relation to the underlying constructs it aimed to measure.

Given that the sample size was greater than 300 participants, factor loadings of.55 were considered acceptable [28]. Following the CFA, modifications were performed according to the first criterion of correlated errors across items with an MI greater than 11 [28]. The fit statistics of the adjusted PCPI-C models are displayed in Table 3.

**Table 3:** Model fit statistics for the adjusted PCPI-C.

		X^2^	*p*	df	X^2^/df	CFI	RMSEA	90% RMSEA	SRMR
**First-order**	Model 1	612,428	.000	125	4,899	.796	.112	.103 –.121	.724
	Model adjusted	286,159	.000	109	2,625	.926	.072	.06 –.08	.052
**Single factor**	Model 1	653,483	.000	135	4,841	.783	.111	.103 –.120	.074
	Model adjusted	269,694	.000	112	2,408	.934	.067	.057 –.078	.049

χ², chi-square; df, degrees of freedom; p, p-value; CFI, Comparative Fit Index; RMSEA, Root Mean Square Error of Approximation; SRMR, Standardized Root Mean Square Residual.

The approach to modify the original structure most respectful of the theoretical model revealed a good adjustment of the model to the study’s sample (Table 3, first-order fit). This evidence of the improved fit due to the adjusted inter-item and inter-construct correlations further prompted us to explore a single-factor structure (Table 3, single-factor fit).

Among the adjusted PCPI-C models, i.e., first-order and single-factor, the best fit to the empirical data was revealed by the single-factor model (best possible fit: x2/df ≈ 1, CFI ≈ 1, RMSEA < .05, SRMR < .08). Specifically, the criteria for the single-factor structure also reflect a good fit, with better indices (x2/df = 2.408, CFI = .934, RMSEA = .067, SRMR = .049).

Considering the best model, i.e., single factor PCPI-C, factor loadings were acceptable except for item 8 (.38). All the other items presented factor loadings greater than .4, with a maximum of .74 (item 13).

The composite reliability for the ‘person-centered processes’ domain improved from the original to the adjusted model in all the constructs for the first-order latent structures. Specifically, when comparing the original and the first-order model, ‘working with the person’s belief and values’ increased from .69 to .75; ‘engaging authentically’ increased from .57 to .74; ‘sharing decision-making’ increased from .74 to .82; ‘being sympathetically present’ increased from .62 to .73; and ‘working holistically’ increased from .71 to .82, which indicates improved internal consistency of the adjusted model.

The average variance extracted (AVE) reveals acceptable convergent validity (i.e., above .5, according to Marôco [28]) for the constructs ‘sharing decision-making’ (AVE = .54) and ‘working holistically’ (AVE = .55), except ‘working with the person’s belief and values’ (AVE = .43), ‘being sympathetically present’ (AVE = .47) and ‘e authentically’ (AVE = .47). Nevertheless, the convergent validity of the construct is still acceptable, as the composite reliability exceeds .6 [35–36].

From the original to the adjusted single-factor model, the composite reliability for the ‘person-centered processes’ domain has also improved, from .90 to .94, which is aligned with the improved internal consistency of the adjusted model and supports the borderline AVE (AVE = .47) for acceptable convergent validity [28,35,36].

## 4 Discussion

For an instrument to be valid and reliable, it is essential to undergo a systematic and thorough process of translation and cultural adaptation [21,37]. In this study, the steps of ISPOR recommendations were followed, including back-translation to ensure the quality of the process. However, translating and culturally adapting the PCPI-C from English to European Portuguese was challenging to preserve the conceptual meanings of the original instrument in terms of language and care concepts. Language is one of the defining elements of a country’s culture. The meaning of words and the interpretation of phenomena depend not only on language but also on a person’s values, education, and life experience. Faced with such a complex and demanding process, the quality of the data resulting from the translated measures depends on the accuracy and quality of the translation. Thus, the translation and cultural adaptation of the PCPI-C followed the recommendations of ISPOR to produce a Portuguese inventory that is conceptually equivalent to the original.

More important than a literal translation is a clear explanation of the basic concepts This approach ensures that the translation captures the conceptual meaning of the items [21]. In the PCPI-C, the translation should be based on colloquial language that is easily understood by respondents, bearing in mind that a population of different ages and levels of education will use the inventory. Key concepts related to care, interpersonal relationships, and individual values may be difficult to translate, given the meaning assigned in different languages, cultures, and healthcare settings [38]. The concepts that generated greater translation difficulty were ‘caring’ and ‘sympathetic presence’ because they are not part of the everyday Portuguese lexicon. To facilitate conceptual equivalence to the original instrument, the instrument developer was included in the process and was able to clarify some uncertainties in the items. The instrument developer is both the author of the underpinning theory and the measurement instrument and is, therefore, the key expert on the concepts and meanings meant to be conveyed in the PCPI-C.

As this is the first translation and validation of the PCPI-C, it is impossible to compare the results obtained with other studies. Nevertheless, the fact that there is an instrument based on the PCPF translated into Portuguese [19] facilitated the translation process, as it allowed for the comparison of the rationale developed throughout the ISPOR stages.

It is important to emphasize that person-centered practice development involves cultural and contextual elements that should not be overlooked [5,39]. Although the PCPI-C covers only one of the four constructs represented in the PCPF, ‘person-centered processes’, data interpretation should integrate the characteristics of the macro context, the care environment, and the healthcare professional’s prerequisites and be combined with other measurements that consider healthcare professionals’ perspectives (i.e., PCPI-S) to obtain a comprehensive view of the person-centered practice [19,30].

In the psychometric evaluation, phase II, the breadth of the sample’s characteristics, namely age and length of hospitalization, can offer distinct perspectives on the experience of care.

The results revealed good adjustment of the first-order model to the study sample, indicating that this instrument can be used as an inventory measure to improve care practices continuously. A single-factor structure concerning the domain of ‘person-centered processes’ was also tested and analyzed. The single-factor fit, with all items contributing to the domain of ‘person-centered processes,’ was revealed to be the best in the empirical data, with the criteria for the single-factor structure also reflecting a good fit, with better indices than the first-order model fit. In this regard, the instrument might allow for assessing the person-centered process as an outcome.

The ceiling effects for some items suggest that the content validity and responsiveness of the PCPI-C can be limited [40] or that the findings are genuinely accurate in representing the PCP in this context. Future studies could determine to what extent the items are sensitive to response bias toward tendentially positive scores.

The use of convenience sampling for psychometric evaluation might limit the generalizability of the findings to a broader population. This study was only performed among public hospital users. The cultural, economic, and linguistic influence and expectations about care might render different results if tested in other fields or backgrounds. To enhance the study’s external validity, future research should consider employing sampling methods that ensure more representative perspectives on person-centered practice across Portuguese service users.

It is also a reminder of the iterative nature of psychometric validation, where empirical findings can inform and refine theoretical models. The analysis of the inventory’s structure yielded different results, highlighting PCA’s exploratory nature and CFA’s theory-driven nature, can help in framing this discussion.

The differences between the PCA and CFA results might reflect the complexity of the theoretical constructs compared to the empirical data patterns. Constructs in psychological theories often have nuanced relationships that might not be perfectly captured by empirical data patterns alone.

The PCA findings suggest a simpler underlying structure (three components) than the theoretical model (five constructs). This discrepancy can arise for several reasons. The overlapping of items with similar loadings onto multiple factors, might indicate that they are not perfectly distinct according to the theoretical model. Moreover, there might be a need to introduce further items to capture the nuances of the five constructs, as some of the components were merged in the PCA.

Ultimately, given its robustness, the simplified model resulting from PCA might be worth exploring as long as it is reconciled with the theoretical principles underlying the inventory. To pursue that aim, a cross-validation procedure with the same analyses with a different sample might provide important contributions to the consistency of the three-component structure. Such a methodological endeavour might also provide a rich opportunity to contribute to the refinement of the theoretical model.

## 5 Conclusion

The PCPF is an internationally recognized theoretical framework for systematically guiding the implementation of person-centered practice in healthcare contexts. With the development and consolidation of the PCPI-S as a valid and reliable instrument for measuring person-centered practice, the need to complement this knowledge by exploring the service user perspective has emerged.

This work contributes with a valid, reliable, and psychometrically good instrument for measuring the person-centered practice based on the PCPF by inquiring about the process through which the person is cared for. The PCPI-C will facilitate monitoring person-centered practice implementation, highlighting changes in a person’s perspective over time. At the same time, it leans toward generating evidence and comparing data with international researchers across settings and clinical areas.

The translation and cultural adaptation process of the PCPI-C from English to European Portuguese was complex and required maintaining the conceptual meaning of the original inventory. The psychometric evaluation revealed two models with good fit indices. Hence, PCPI-C users must consider the purpose for which the instrument will be used to select the most appropriate measurement model, i.e., process evaluation as an outcome or as an inventory measure for continuous improvement.

## Supporting information

S1 Appendix Cognitive interview script. (DOCX)

S1 Table Correlation matrix. (DOCX)

S2 Table Rotated Factor Matrix. (DOCX)
