# Supplementary material for: Portuguese translation, cultural adaptation, and validation of the Person-Centered Practice Inventory – Care
Source: PLoS One. 2025 May 28;20(5):e0324286. doi: 10.1371/journal.pone.0324286 (PMC12118862; doi:10.1371/journal.pone.0324286)
Supplement: S1 Appendix — (DOCX) [file pone.0324286.s001.docx]

1. **Cognitive interview protocol**

The most effective cognitive interviewing methods consist of a combination of predefined questions specifically designed to obtain detailed information on the aspects under analysis, such as verbal probing, and more flexible ones, in which interviewees are asked to actively verbalize their thoughts while responding to the items tested, such as think-aloud technique [1-3].

Therefore, the guidelines for the interviews are as follows:

1.1 The researcher will interview in person, as this allows for observing nonverbal expressions and favors a natural interaction between the interviewer and the participant [4].

1.2 The interviews will be audio recorded.

1.3 Before the interview, some clarifications will be given:

- The aim of the interview is not to obtain answers from the inventory; it is to identify difficulties of interpretation in questions and understand the reasoning that leads to the answer or the interpretation problems encountered;

- The participant is free to criticize the inventory at will;

- The researcher is not the questionnaire's author; he or she translates it to eliminate constraints on the participant [4].

1.4 The participants were invited to complete the questionnaire in its entirety in paper or digital format. To support their answers, they are instructed to recall an interaction episode with health professionals that was significant for them or occurred recently.

1.5 The participants are asked to comment on any aspect that has been omitted or raises doubts about it.

1.6 The researcher used the think-aloud technique and provided instructions such as "Please say what you thought to agree/disagree with this statement," "I noticed you hesitated. Was it difficult to answer this statement?" and "Explain the thought that led you to your answer." At this stage, the researcher does not influence the participant's answers and focuses on understanding the reasoning inherent in each item [4].

1.7 Afterwards, specific questions will be asked about the questionnaire using the verbal probing technique, which is applied retrospectively so that the participants can express their understanding of each statement more clearly and thoroughly. The questions will be of the following type: "State in your own words the content of the statement," "How did you understand this statement?" and "What does the concept mean to you?"

1.8 A script of guiding questions was drawn for the verbal probing phase for each item (Table 1). However, other questions that arise spontaneously during the interview may be used.

Table 1 – Verbal probing guide

| **Items** | **Questions** |
| --- | --- |
| 1. Healthcare professionals make an effort to understand what is important to me | - Who do you think of when you read “healthcare professionals”?  - What do you mean by “what is important to me”?  - What do healthcare professionals strive to understand? |
| 2. Healthcare professionals use my personal experiences to build a relationship with me | - When you think of “personal experience”, what are you thinking of?  - What do you mean by “building a relationship”?  - How do you think your personal experience was useful for healthcare professionals? |
| 3. Healthcare professionals involve me in making decisions about my care  OR Healthcare professionals involve me in decision-making about my care* | - What do you mean by "being involved in decisions"?  - Can you give an example of a situation in which you were involved in decision-making about your care? |
| 4. Healthcare professionals take my home context into consideration in satisfying my care needs | - What do you mean by "home context"?  - How important is it to include the home context in meeting care needs?  - How do you think your home context was useful for your care? |
| 5. Healthcare professionals give me their full attention when they are with me | - What do you mean by "giving their full attention"?  - Can you share a time when you felt you received "full attention"? |
| 6. I feel free to say healthcare professionals what is important to me | - What do you mean by "what's important to me"? - Can you share an example of a conversation with health professionals in which you shared what was important to you? |
| 7. I feel capable to share my perceptions about my care experience with healthcare professionals  OR I feel capable to give my opinion about my care experience with healthcare professionals* | - What do you mean by "feeling capable"?  - What could limit your ability to give your perception?  - What could limit your ability to give your opinion?  - What is the difference between perception and opinion for you? |
| 8. Healthcare professionals ask me questions about my life | - What do you mean by being asked "questions about your life"?  - Can you say this sentence in your own words?  - Is there a word that the person would use to encompass these aspects that the expression "about my life" led you to list? |
| 9. Healthcare professionals establish a connection with me as a person | - How do you interpret the word "connection"?  - Say this statement in your own words. |
| 10. Healthcare professionals ask me if I have all the information I need | - What kind of information is implied in this statement?  - What information is important for you to have? |
| 11. When we disagree about my care, healthcare professionals try to reach a consensus | - What do you mean by "reaching a consensus"?  - Can you think of a time when you disagreed with the opinion of a healthcare professional? |
| 12. In caring for me, healthcare professionals use what they know about me as a person | - Explain this statement.  - What can healthcare professionals know about you as a person? |
| 13. I feel that I’m cared for | - What do you mean by “being cared for"?  - Give an example of a situation in which you felt “cared for”. |
| 14. Healthcare professionals respond with empathy when I am upset or unhappy OR  Healthcare professionals respond with compassion when I am upset or unhappy* | - What do you mean by "responding with empathy/compassion"?  - Can you remember a time when you felt this way? How did the healthcare professionals react?  - Can you respond to the item even if you have not had this experience?  - What do you expect from a healthcare professional who responds with empathy/compassion?  - What is the difference between compassion and emphaty for you? |
| 15. Healthcare professionals help me to express my concerns about my treatment and care | - Give an example of how healthcare professionals can help you "express your concerns". |
| 16. Healthcare professionals listen to me and hear what I have to say about care | - Explain what "listen to me and hear what I have to say" means to you. |
| 17. Healthcare professionals understand my family circumstances when they take care of me | - What are "family circumstances"?  - How did the healthcare professionals knowledge of your family circumstances influence the care they gave you? |
| 18. Healthcare professionals help me to define realistic goals | - What do you consider to be "realistic goals"?  - How have healthcare professionals helped you define your goals? |

**Note:** the items marked with an * are the original ones, tested and changed during Round 1.

1.9 As the interviews occur, the data discussed should be integrated into the following ones to confront the answers and open up other perspectives on the item [5].

1.10 It should be validated with the participants whether the interviewer's interpretation coincides with the information they have transmitted [4].

1.11 Items considered challenging to interpret will be identified, and those that the participants have difficulty understanding will be selected for adjustment. If the participant asks to repeat the wording, answer options, and/or provide an example, they will be considered to have difficulty understanding the item [6].

1.12 A first round of interviews will be conducted, focusing on aspects of interpreting general concepts [4]. If necessary, adjustments will be made to the flagged items, and a second round will be conducted. This round should focus on validating questions of spelling, structure, and grammatical form.

**References**

1. Betty P, Willis G. Research synthesis: the practice of cognitive interviewing. Public Opinion Quarterly. 2007; 71:2; 287–311. doi:10.1093/poq/nfm006
2. Willis GB. Cognitive interviewing revisited: A useful technique, in theory? In Methods for testing and evaluating survey questionnaires. 2004; 23-43. doi:[10.1002/0471654728.ch2](http://dx.doi.org/10.1002/0471654728.ch2)
3. Willis G, Artino A. What Do Our Respondents Think We’re Asking? Using Cognitive Interviewing to Improve Medical Education Surveys. Journal of Graduate Medical Education. 2013; 353-356. doi:10.4300/JGME-D-13-00154.1
4. Willis G. Cognitive Interviewing: A “How To” Guide. Research Triangle Institute. 1999 <http://appliedresearch.cancer.gov/areas/cognitive/interview.pdf> Accessed 10 jan 2022
5. Thorne SE. Interpretive description: qualitative research for applied practice. Second edition. New York; London: Routledge. 2016.
6. Vaz D. Silva D. Campos DS. Antunes A. Magalhães L. Furtado S. Aplicação de entrevistas cognitivas para produção de versões brasileiras de instrumentos de avaliação da prática centrada na família. Rev Ter Ocup Univ São Paulo. 2018; 29:1; 41-49. doi: 10.11606/issn.2238-6149.v29i1p41-49
