# Supplementary material for: Portuguese translation, cultural adaptation, and validation of the Person-Centered Practice Inventory – Care
Source: PLoS One. 2025 May 28;20(5):e0324286. doi: 10.1371/journal.pone.0324286 (PMC12118862; doi:10.1371/journal.pone.0324286)
Supplement: S1 Table — (DOCX) [file pone.0324286.s002.docx]

| **S1 Table - Correlation matrix.** | | | | | | | | | | | | | | | | | | | |
| --- | --- | --- | --- | --- | --- | --- | --- | --- | --- | --- | --- | --- | --- | --- | --- | --- | --- | --- | --- |
|  | | I1 | I2 | I3 | I4 | I5 | I6 | I7 | I8 | I9 | I10 | I11 | I12 | I13 | I14 | I15 | I16 | I17 | I18 |
| I1 | r |  | ,403** | ,428** | ,380** | ,620** | ,476** | ,356** | ,141* | ,421** | ,262** | ,190** | ,246** | ,566** | ,358** | ,396** | ,372** | ,339** | ,331** |
|  | Sig. (2-tailed) |  | 0,0 | 0,000 | 0,000 | 0,000 | 0,000 | 0,000 | 0,013 | 0,000 | 0,000 | 0,001 | 0,000 | 0,000 | 0,000 | 0,000 | 0,000 | 0,000 | 0,000 |
|  | N |  | 312 | 312 | 312 | 312 | 312 | 312 | 312 | 312 | 312 | 312 | 312 | 312 | 312 | 312 | 312 | 312 | 312 |
| I2 | r |  |  | ,369** | ,419** | ,343** | ,292** | ,329** | ,275** | ,436** | ,292** | ,151** | ,281** | ,332** | ,348** | ,324** | ,296** | ,204** | ,290** |
|  | Sig. (2-tailed) |  |  | 0,000 | 0,000 | 0,000 | 0,000 | 0,000 | 0,000 | 0,000 | 0,000 | 0,008 | 0,000 | 0,000 | 0,000 | 0,000 | 0,000 | 0,000 | 0,000 |
|  | N |  |  | 312 | 312 | 312 | 312 | 312 | 312 | 312 | 312 | 312 | 312 | 312 | 312 | 312 | 312 | 312 | 312 |
| I3 | r |  |  |  | ,480** | ,394** | ,443** | ,423** | ,228** | ,316** | ,461** | ,351** | ,281** | ,459** | ,329** | ,429** | ,369** | ,342** | ,400** |
|  | Sig. (2-tailed) |  |  |  | 0,000 | 0,000 | 0,000 | 0,000 | 0,000 | 0,000 | 0,000 | 0,000 | 0,000 | 0,000 | 0,000 | 0,000 | 0,000 | 0,000 | 0,000 |
|  | N |  |  |  | 312 | 312 | 312 | 312 | 312 | 312 | 312 | 312 | 312 | 312 | 312 | 312 | 312 | 312 | 312 |
| I4 | r |  |  |  |  | ,408** | ,364** | ,270** | ,408** | ,322** | ,418** | ,347** | ,268** | ,473** | ,358** | ,393** | ,378** | ,493** | ,427** |
|  | Sig. (2-tailed) |  |  |  |  | 0,000 | 0,000 | 0,000 | 0,000 | 0,000 | 0,000 | 0,000 | 0,000 | 0,000 | 0,000 | 0,000 | 0,000 | 0,000 | 0,000 |
|  | N |  |  |  |  | 312 | 312 | 312 | 312 | 312 | 312 | 312 | 312 | 312 | 312 | 312 | 312 | 312 | 312 |
| I5 | r |  |  |  |  |  | ,582** | ,458** | ,227** | ,482** | ,269** | ,184** | ,366** | ,620** | ,391** | ,456** | ,433** | ,305** | ,397** |
|  | Sig. (2-tailed) |  |  |  |  |  | 0,000 | 0,000 | 0,000 | 0,000 | 0,000 | 0,001 | 0,000 | 0,000 | 0,000 | 0,000 | 0,000 | 0,000 | 0,000 |
|  | N |  |  |  |  |  | 312 | 312 | 312 | 312 | 312 | 312 | 312 | 312 | 312 | 312 | 312 | 312 | 312 |
| I6 | r |  |  |  |  |  |  | ,635** | ,159** | ,494** | ,298** | ,231** | ,262** | ,537** | ,296** | ,450** | ,437** | ,268** | ,353** |
|  | Sig. (2-tailed) |  |  |  |  |  |  | 0,000 | 0,005 | 0,000 | 0,000 | 0,000 | 0,000 | 0,000 | 0,000 | 0,000 | 0,000 | 0,000 | 0,000 |
|  | N |  |  |  |  |  |  | 312 | 312 | 312 | 312 | 312 | 312 | 312 | 312 | 312 | 312 | 312 | 312 |
| I7 | r |  |  |  |  |  |  |  | ,220** | ,447** | ,374** | ,286** | ,249** | ,451** | ,341** | ,399** | ,434** | ,285** | ,436** |
|  | Sig. (2-tailed) |  |  |  |  |  |  |  | 0,000 | 0,000 | 0,000 | 0,000 | 0,000 | 0,000 | 0,000 | 0,000 | 0,000 | 0,000 | 0,000 |
|  | N |  |  |  |  |  |  |  | 312 | 312 | 312 | 312 | 312 | 312 | 312 | 312 | 312 | 312 | 312 |
| I8 | r |  |  |  |  |  |  |  |  | ,343** | ,422** | ,177** | ,302** | ,217** | ,287** | ,249** | ,236** | ,446** | ,419** |
|  | Sig. (2-tailed) |  |  |  |  |  |  |  |  | 0,000 | 0,000 | 0,002 | 0,000 | 0,000 | 0,000 | 0,000 | 0,000 | 0,000 | 0,000 |
|  | N |  |  |  |  |  |  |  |  | 312 | 312 | 312 | 312 | 312 | 312 | 312 | 312 | 312 | 312 |
| I9 | r |  |  |  |  |  |  |  |  |  | ,244** | ,177** | ,475** | ,429** | ,372** | ,413** | ,490** | ,291** | ,303** |
|  | Sig. (2-tailed) |  |  |  |  |  |  |  |  |  | 0,000 | 0,002 | 0,000 | 0,000 | 0,000 | 0,000 | 0,000 | 0,000 | 0,000 |
|  | N |  |  |  |  |  |  |  |  |  | 312 | 312 | 312 | 312 | 312 | 312 | 312 | 312 | 312 |
| I10 | r |  |  |  |  |  |  |  |  |  |  | ,304** | ,185** | ,294** | ,313** | ,334** | ,263** | ,414** | ,436** |
|  | Sig. (2-tailed) |  |  |  |  |  |  |  |  |  |  | 0,000 | 0,001 | 0,000 | 0,000 | 0,000 | 0,000 | 0,000 | 0,000 |
|  | N |  |  |  |  |  |  |  |  |  |  | 312 | 312 | 312 | 312 | 312 | 312 | 312 | 312 |
| I11 | r |  |  |  |  |  |  |  |  |  |  |  | ,153** | ,293** | ,307** | ,311** | ,265** | ,444** | ,289** |
|  | Sig. (2-tailed) |  |  |  |  |  |  |  |  |  |  |  | 0,007 | 0,000 | 0,000 | 0,000 | 0,000 | 0,000 | 0,000 |
|  | N |  |  |  |  |  |  |  |  |  |  |  | 312 | 312 | 312 | 312 | 312 | 312 | 312 |
| I12 | r |  |  |  |  |  |  |  |  |  |  |  |  | ,328** | ,364** | ,266** | ,270** | ,316** | ,302** |
|  | Sig. (2-tailed) |  |  |  |  |  |  |  |  |  |  |  |  | 0,000 | 0,000 | 0,000 | 0,000 | 0,000 | 0,000 |
|  | N |  |  |  |  |  |  |  |  |  |  |  |  | 312 | 312 | 312 | 312 | 312 | 312 |
| I13 | r |  |  |  |  |  |  |  |  |  |  |  |  |  | ,431** | ,561** | ,568** | ,315** | ,388** |
|  | Sig. (2-tailed) |  |  |  |  |  |  |  |  |  |  |  |  |  | 0,000 | 0,000 | 0,000 | 0,000 | 0,000 |
|  | N |  |  |  |  |  |  |  |  |  |  |  |  |  | 312 | 312 | 312 | 312 | 312 |
| I14 | r |  |  |  |  |  |  |  |  |  |  |  |  |  |  | ,509** | ,424** | ,457** | ,478** |
|  | Sig. (2-tailed) |  |  |  |  |  |  |  |  |  |  |  |  |  |  | 0,000 | 0,000 | 0,000 | 0,000 |
|  | N |  |  |  |  |  |  |  |  |  |  |  |  |  |  | 312 | 312 | 312 | 312 |
| I15 | r |  |  |  |  |  |  |  |  |  |  |  |  |  |  |  | ,643** | ,394** | ,522** |
|  | Sig. (2-tailed) |  |  |  |  |  |  |  |  |  |  |  |  |  |  |  | 0,000 | 0,000 | 0,000 |
|  | N |  |  |  |  |  |  |  |  |  |  |  |  |  |  |  | 312 | 312 | 312 |
| I16 | r |  |  |  |  |  |  |  |  |  |  |  |  |  |  |  |  | ,358** | ,424** |
|  | Sig. (2-tailed) |  |  |  |  |  |  |  |  |  |  |  |  |  |  |  |  | 0,000 | 0,000 |
|  | N |  |  |  |  |  |  |  |  |  |  |  |  |  |  |  |  | 312 | 312 |
| I17 | r |  |  |  |  |  |  |  |  |  |  |  |  |  |  |  |  |  | ,601** |
|  | Sig. (2-tailed) |  |  |  |  |  |  |  |  |  |  |  |  |  |  |  |  |  | 0,000 |
|  | N |  |  |  |  |  |  |  |  |  |  |  |  |  |  |  |  |  | 312 |
| I18 | r |  |  |  |  |  |  |  |  |  |  |  |  |  |  |  |  |  |  |
|  | Sig. (2-tailed) |  |  |  |  |  |  |  |  |  |  |  |  |  |  |  |  |  |  |
|  | N |  |  |  |  |  |  |  |  |  |  |  |  |  |  |  |  |  |  |
| ** Correlation is significant at the 0.01 level (2-tailed). | | | | | | | | | | | | | | | | | | | |
| * Correlation is significant at the 0.05 level (2-tailed).  r, Pearson correlation. | | | | | | | | | | | | | | | | | | | |
