# Supplementary material for: Portuguese translation, cultural adaptation, and validation of the Person-Centered Practice Inventory – Care
Source: PLoS One. 2025 May 28;20(5):e0324286. doi: 10.1371/journal.pone.0324286 (PMC12118862; doi:10.1371/journal.pone.0324286)
Supplement: S2 Table — (DOCX) [file pone.0324286.s003.docx]

| **Rotated Factor Matrix^a^** | | | |
| --- | --- | --- | --- |
|  | Factor | | |
|  | 1 | 2 | 3 |
| [Item 13] | ,721 |  |  |
| [Item 6] | ,711 |  |  |
| [Item 5] | ,689 |  |  |
| [Item 1] | ,620 |  |  |
| [Item 7] | ,564 |  |  |
| [Item 16] | ,553 |  |  |
| [Item 15] | ,552 | ,422 |  |
| [Item 3] | ,476 | ,434 |  |
| [Item 17] |  | ,723 |  |
| [Item 18] |  | ,631 |  |
| [Item 10] |  | ,556 |  |
| [Item 4] |  | ,535 |  |
| [Item 8] |  | ,498 | ,465 |
| [Item 11] |  | ,483 |  |
| [Item 14] |  | ,438 |  |
| [Item 9] | ,477 |  | ,655 |
| [Item 12] |  |  | ,495 |
| [Item 2] |  |  |  |
| Extraction Method: Principal Axis Factoring.  Rotation Method: Varimax with Kaiser Normalization.^a^ | | | |
| a. Rotation converged in 8 iterations. | | | |
